# Effect of Oil on Cellulose Dissolution in the Ionic Liquid 1-Butyl-3-methyl Imidazolium Acetate

**DOI:** 10.1021/acsomega.2c04311

**Published:** 2022-10-12

**Authors:** Katherine S. Lefroy, Brent S. Murray, Michael E. Ries

**Affiliations:** † School of Food Science and Nutrition, University of Leeds, LeedsLS2 9JT, U.K.; ‡ School of Physics and Astronomy, University of Leeds, LeedsLS2 9JT, U.K.

## Abstract

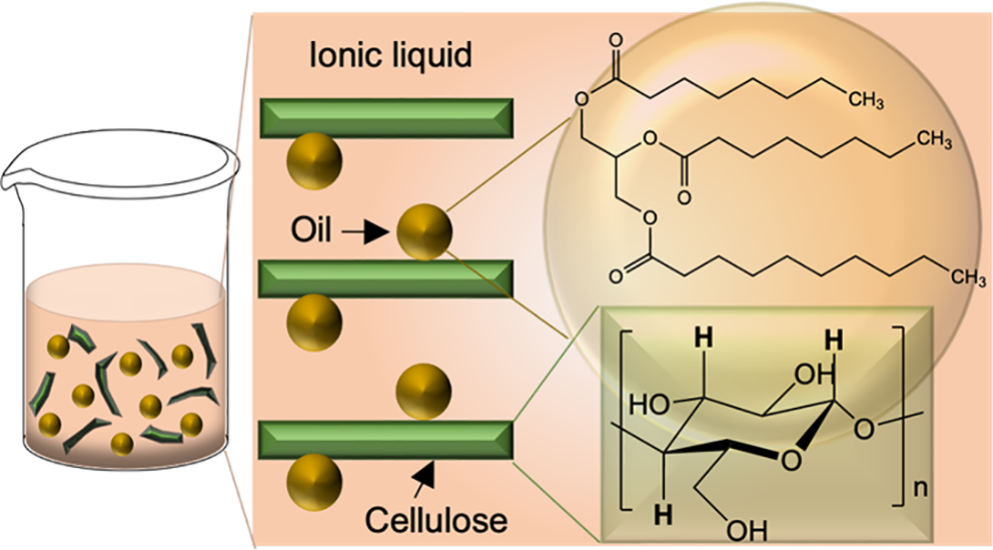

While ionic liquids (ILs) are well known to be excellent solvents for cellulose, the exact mechanism of dissolution has been a much disputed topic in recent years and is still not completely clear. In this work, we add to the current understanding and highlight the importance of hydrophobic interactions, through studying cellulose dissolution in mixtures of 1-butyl-3-methyl imidazolium acetate (BmimAc) and medium-chain triglyceride (MCT) oil. We demonstrate that the order in which constituents are mixed together plays a key role, through nuclear magnetic resonance (NMR) spectroscopic analysis. When small quantities of MCT oil (0.25–1 wt %) were introduced to BmimAc before cellulose, the effect on BmimAc chemical shift values was much more significant compared to when the cellulose was dissolved first, followed by oil addition. Rheological analysis also showed small differences in the viscosities of oil–cellulose–BmimAc solutions, depending on the order the constituents were added. On the other hand, no such order effect on the NMR results was observed when cellulose was replaced with cellobiose, suggesting that this observation is unique to the macromolecule. We propose that a cellulose–oil interaction develops but only when the cellulose structure has a sufficient degree of order and not when the cellulose is molecularly dispersed, since the hydrophobic cellulose plane is no longer intact. In all cases, cellulose–BmimAc–oil solutions were stable for at least 4 months. To our knowledge, this is the first work that investigates the effect of oil addition on the dissolving capacity of BmimAc and highlights the need for further re-evaluation of accepted mechanisms for cellulose dissolution in ILs.

## Introduction

Ionic liquids (ILs) have provided a major breakthrough in the dissolution, modification, and general functionalization of biomass and its components, including cellulose, providing a non-toxic and potentially “greener” pathway to making useful materials.^1,2^ Since their “rediscovery”^3^ in 2002 by Swatloski et al.,^4^ ILs have been used extensively in research to dissolve cellulose for different purposes, for example, for packaging, novel materials, composites, and food ingredients.^2,5−7^ Imidazolium-based ILs are commonly favored since they are usually liquid at room temperature, relatively hydrophilic,^8^ and thus have high cellulose-dissolving capabilities,^9^ (up to 25–27 wt % in some cases).^4,8,10^ The mechanism of cellulose dissolution in imidazolium-based ILs should be understood to design more efficient, biodegradable, and “greener” ILs;^11,12^ however, the role of hydrophilic and hydrophobic interactions is still heavily debated.

The majority of cellulose–IL dissolution studies highlight the importance of the interaction between the anion and the cellulose hydroxyl groups,^4,13,14^ and it is often cited that inter- and intramolecular H-bonds between cellulose chains must be broken to achieve dissolution.^15,16^ However, the role of the cation is more disputed, and the significance of hydrophobic interactions is often not addressed.^17,18^ On one hand, it is argued that the cation also participates in H-bonding with cellulose and therefore very bulky cations cannot penetrate and reach the hydroxyl groups,^19,20^ making the IL less effective for cellulose dissolution. Equally, Lu et al. focused on H-bonding, claiming that the structure of the cation is important and the strength of the anion–cation H-bond must also be taken into account.^11^ On the other hand, it has been proposed that the cation neutralizes the negatively charged H-bonded anion–cellulose complex, which leads to increased steric repulsion in the complex and the development of a hydrophobic interaction between the comparatively bulky cation and the hydrophobic regions on cellulose chains.^21,22^ Furthermore, molecular dynamics (MD) simulations have revealed that hydrophobic interactions are responsible for close contact between the cation and cellulose, exposing a stacking interaction between imidazolium cations and cellulose pyranose rings.^23,24^

Due to the amphiphilic nature of cellulose molecules, it is necessary to consider the role of both hydrophilic and hydrophobic interactions^25^ when designing ILs for cellulose dissolution. An effective solvent should have both polar and nonpolar regions;^26^ however, ILs employed in cellulose dissolution are usually water-miscible and often described only as hydrophilic,^27^ a property controlled by the anion.^28^ Since cellulose is insoluble in water, it is evident that the cellulose-dissolving ability of ILs cannot be solely based on H-bonding and, upon closer inspection of the cation, the best candidates in fact display strong structural asymmetry.^29^ The amphiphilic nature of the cation influences its hydrophobicity and therefore may play a role in facilitating cellulose dissolution.^18^

Some of the major drawbacks associated with cellulose–IL processing on a large scale are the relatively high cost and viscosity of ILs,^1,30−34^ the latter of which can become very high when significant amounts of cellulose are dissolved.^35^ Furthermore, the presence of small impurities such as halides and imidazoles (from the preparation of the IL)^9,36^ may also have some effect and even lead to hydrolysis of cellulose and other side reactions,^37−39^ while the presence of water can completely disrupt the H-bond network of cation–anion pairs and dramatically alter the cellulose-dissolving ability.^40−45^ Water contamination has been shown to trigger phase separation^31^ and can affect cellulose dissolution at concentrations as low as 1 wt %,^4^ which is particularly problematic in imidazolium-based ILs due to their highly hygroscopic nature.^41^ However, Fendt et al. suggested that if water is present in very small quantities, it may reduce the viscosity of some ILs and act as a cosolvent, thus improving cellulose dissolution.^46^

More significant lowering of viscosities has been reported with polar aprotic cosolvents, such as dimethyl sulfoxide (DMSO) or dimethyl formamide (DMF), which can be added in greater quantities than water to ILs either before or after cellulose dissolution.^47−49^ Efficient cosolvents can reduce the volume fraction of IL required, lowering the processing cost, and may even enhance IL properties by increasing the cellulose dissolution speed and dissolving capacity.^47,50−52^ Therefore, binary cosolvent/IL mixtures have received much attention in recent years due to their more promising potential for industrial use compared to pure ILs. If the cosolvent is added before dissolution, it is sometimes described as a “pre-swelling” stage during which the cosolvent can begin to penetrate between cellulose layers and disrupt the highly ordered structure.^53^ Furthermore, pre-treatment of biomass with cosolvents can remove lignin and hemicelluloses (which can act as a barrier to cellulose dissolution), increasing the cellulose surface area and further facilitating dissolution in the IL.^54^ In general, solvents which are weak hydrogen bond donors (HBDs) and have high values for polarity, basicity, and dipolarity/polarizability are miscible with hydrophilic ILs and therefore considered efficient cosolvents,^27^ while solvents displaying good HBD ability can have the opposite effect.^27^ Most studies therefore focus exclusively on polar cosolvents, while less attention is given to nonpolar cosolvents.

Understanding the effect of both polar and nonpolar species on ILs is of fundamental importance, since different impurities may be present in their applications as lubricants,^55^ biocatalysts,^56^ electrolytes,^57^ and coolants.^58^ In terms of cellulose dissolution, species of different polarities may equally affect the process due to the amphiphilic character of the solvent and the solute. Broadly speaking, the most efficient ILs for cellulose dissolution possess cations with dipolar character (often achieved by a heterocyclic aromatic ring) and anions which are non-bulky and weakly hydrophobic (in order to provide several H-bond acceptor sites).^59^ It has previously been demonstrated that miscibility between an IL and a cosolvent is largely determined by the ratio of alkyl chain lengths for a protic IL and a non-polar additive,^60^ while certain ILs may support amphiphilic self-assembly.^61^ However, aprotic ILs capable of dissolving cellulose generally have a very limited miscibility with hydrophobic reagents and cellulose derivatives and therefore adding a small quantity of non-polar cosolvent may allow one to tune the IL properties and potentially provide a route for the preparation of more hydrophobic cellulose materials.^27^ This could have advantages in the functionalization of cellulose from ILs, allowing further manipulation of cellulose properties. While some detailed studies on nonpolar cosolvent/IL binary mixtures have been conducted, to our knowledge, these have been restricted to ILs with rather hydrophobic anions (e.g., Tf_2_N^–^ and PF_6_^–^) which are not suitable solvents for cellulose.^62,63^

In this work, we have used a combination of ultraviolet–visible (UV–vis) and nuclear magnetic resonance (NMR) spectroscopy to investigate the interactions present in cellulose, 1-butyl-3-methyl imidazolium acetate (BmimAc), and medium-chain triglyceride (MCT) oil mixtures. We present experiments analyzing the effects that low concentrations of nonpolar solvent have on cellulose dissolution in ILs. A relatively ‘polar’ oil was selected with some hydrophilic character, in order to maximize the possibility of interaction between the amphiphilic cellulose/BmimAc and the oil. Oil–BmimAc solutions with/without cellulose were analyzed, which indicated a preferential interaction between cellulose and oil as opposed to oil and BmimAc, providing further strong evidence for the structural anisotropy of cellulose. However, we also show that amphiphilicity changes depending on the state of the cellulose in solution and its degree of order. To our knowledge, this is the first experimental study of its kind investigating the effect of nonpolar solvents on cellulose–IL dissolution. Most notably, the order in which cellulose and oil were added to BmimAc was found to have a significant effect on the resultant properties of the solution both microscopically and macroscopically.

## Materials and Methods

BmimAc (≥95% purity) was obtained from Sigma-Aldrich, and cellulose powder (Vitacel L 00) was supplied by Mondele̅z International. Full details of the cellulose powder including the degree of polymerization are provided in the Supporting Information (Table S1). Cellobiose powder was obtained from BioServ UK limited. MCT oil Miglyol 812 (caprylic/capric triglycerides^64^) with a density of 0.945 g mL^–1^ at 20 °C was obtained from Cremer Oleo GmbH & Co (Germany).

### UV–Visible Spectroscopy

UV–vis absorbance spectra were recorded on a Specord 210 Plus spectrophotometer (Analytik Jena) with a slit width of 1 nm at *T* = 298 K. All samples were pipetted into quartz cuvettes (ca. 3 mL) with a path length of 1 cm. The absorbance was scanned over a range of wavelengths (180–800 nm).

### ^1^H NMR (High-Field) Spectroscopy

^1^H NMR spectra were recorded on a Bruker AVANCE II NMR spectrometer, operating at a ^1^H resonance frequency of 400 MHz. All measurements were performed at a temperature of 298 K. Each sample was pipetted into 5 mm NMR tubes and a capillary loaded with DMSO-*d*_6_ was added as a reference, so as to avoid any contact between DMSO-*d*_6_ and the samples. Each spectrum was calibrated to the ^1^H external reference of the residual proton in DMSO-*d*_6_, at 2.5 ppm.^65^ All samples were prepared in a glovebox (Braun) to minimize water contamination. All samples had significantly less than 0.5 wt % water.

### Rheological Measurements

Steady-state shear viscosity measurements of cellulose–BmimAc–oil mixtures were conducted on an Anton Paar MCR 302 (Anton Paar GmbH, Graz, Austria) rheometer using a circular cone-plate geometry with a diameter of 50 mm and an angle of 2°. The temperature was fixed at a constant 25 °C throughout all of the measurements, controlled by a water bath temperature control unit and a Peltier hood. A pre-shear at 1 s^–1^ was included for 3 min, allowing adequate heating throughout the sample, before the viscosity was measured between 0.01 and 1000 s^–1^. All measurements were repeated three times.

### Optical Microscopy

Cellulose–BmimAc–oil mixtures were analyzed using a light microscope (Nikon, SMZ-2T, Japan), equipped with a digital camera (Leica MC120 HD) and 10×/20× lenses. A drop of solution was placed on a welled slide and covered with a coverslip (0.17 mm thickness). Images were processed using the image analysis software ImageJ.

## Results and Discussion

### Determining the Miscibility of BmimAc and Oil, with and without Cellulose (UV–Vis)

**Figure 1:**
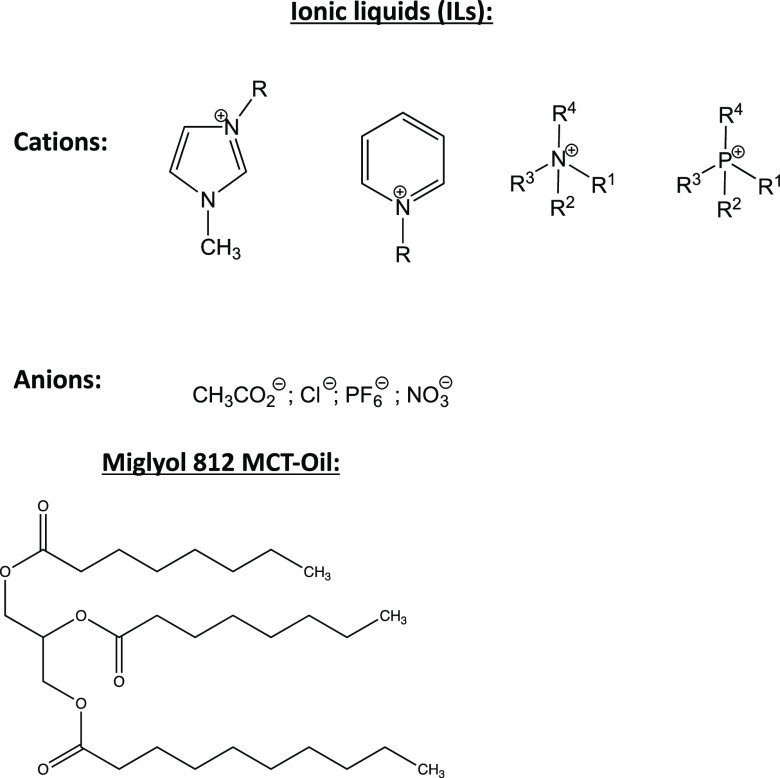
Chemical structures of the cations and anions of some common ILs used for dissolving cellulose and MCT oil (capric/caprylic triglycerides).

The miscibility of pure BmimAc and oil was analyzed and compared to cellulose–BmimAc solutions and oil using UV–vis and ^1^H NMR. As previously described in the introduction, the structural asymmetry of ILs is key to their cellulose-dissolving capabilities, since cellulose itself has structural anisotropy.^25^ The cationic structural asymmetry also lowers the IL viscosity, making it a more effective cellulose solvent.^14^Figure 1 shows the chemical structures of some common ILs used for dissolving cellulose, each consisting of a bulky cation, and MCT oil. It is reasoned that there is potential for some hydrophobic association between the amphiphilic cation and nonpolar molecules, and therefore, a relatively polar oil was selected in order to maximize the chance of IL–oil interaction and miscibility. Furthermore, we have previously reported that oils with higher polarities were most successful in producing “oily” cellulose stabilizers for water-in-oil (W/O) emulsions, most likely because of their ability to form some kind of hydrophilic interaction with cellulose during coagulation.^7^ Other less-polar oils, for example, tetradecane, were not able to produce stable W/O emulsions and the majority of oil appeared to be washed away during coagulation, rather than interacting with cellulose.

**Figure 2:**
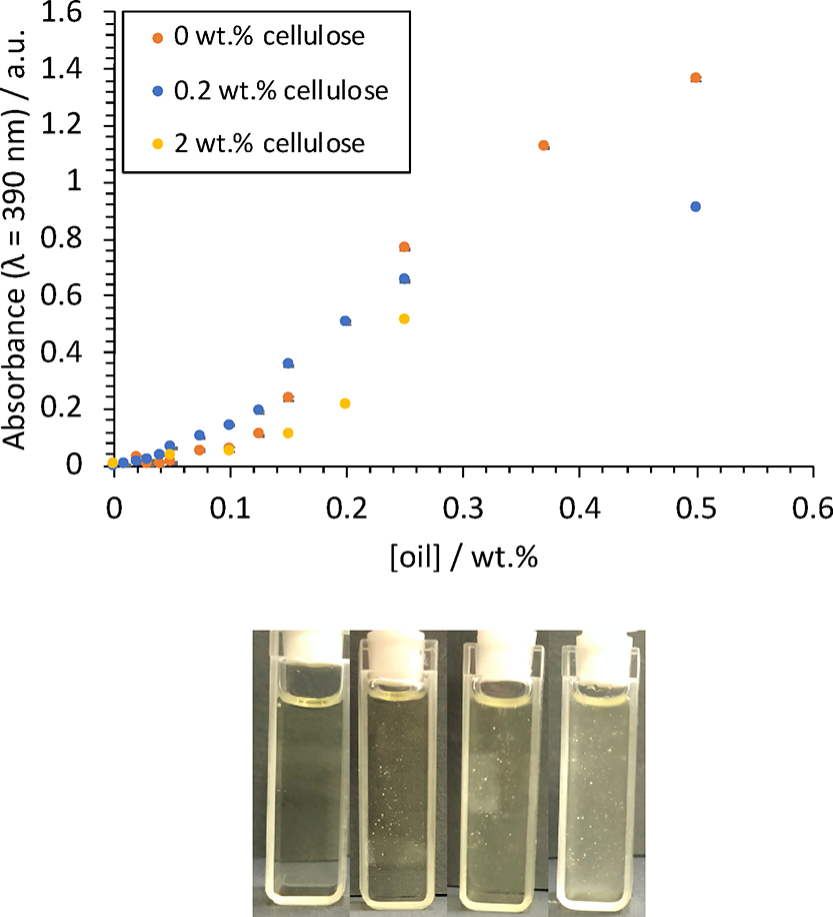
Absorbance at λ = 390 nm for BmimAc/MCT oil mixtures as a function of oil concentration ([oil]), without cellulose (orange circle), with 0.2 wt % Vitacel L 00 cellulose (blue circle), and with 2 wt % Avicel cellulose (yellow circle). Error bars are shown, but some may be hidden by the symbol. Image below shows the appearance of 0.2 wt % cellulose in BmimAc/MCT oil solutions with 0, 0.05, 0.2, and 0.5 wt % oil (from left to right).

First, in order to determine the maximum solubility of oil in BmimAc both with and without cellulose, solutions were prepared with very small amounts of MCT oil either without cellulose or with cellulose dissolved initially before the addition of oil. Solutions were analyzed using UV–vis spectroscopy, first by scanning the absorbance wavelengths (λ) and then selecting a set wavelength of 390 nm. Although this was not a “maximum” absorbance peak which is expected to be approximately 290 nm for ILs with a (Bmim)^+^ cation,^66^ the absorbance was very noisy at lower wavelengths most probably due to instrument limitations. While the reference sample (pure BmimAc) has lower transmission at this wavelength, differences between solutions which appeared optically identical could be observed.^67^ Full absorbance spectra for BmimAc–oil solutions with an oil concentration ([oil]) of 0.25 wt % and with cellulose concentrations ([cellulose]) of 0, 0.2, and 2 wt % cellulose can be found in Figure S1. Figure 2 (orange circles) shows the absorbance spectra at a single wavelength (λ = 390 nm) for 0, 0.2, and 2 wt % cellulose in BmimAc–oil solutions, for which the absorbance was measured over a range of [oil] = 0–1 wt %. All solutions became very turbid after [oil] ≃ 0.5 wt % (both with and without cellulose, image below Figure 2), making the measurements less reliable due to scattering of the beam. Therefore, this was determined to be the approximate limit of “solubility”, and [oil] > 0.5 wt % was not measured using this technique. Data points for the 2 wt % cellulose–BmimAc solution at [oil] > 0.3 wt % were also omitted due to their high viscosity and thus complications with introducing them to the cuvette.

At low concentrations of oil for all solutions, the absorbance increased steadily with increasing [oil]. A sharper increase in absorbance was then observed for [oil] > ca. 0.12 wt %, particularly for the cellulose-free solution, where it rose from 0.505 to 1.37 between [oil] = 0.2 and 0.5 wt %, respectively. This indicates that the MCT oil may be dispersing rather than dissolving at concentrations exceeding 0.25 wt %, and therefore, only a very small amount of oil is truly miscible with the pure IL. Interestingly, when just 0.2 wt % cellulose was dissolved in the solution, the increase in absorbance was less drastic, rising from 0.503 to 0.905 between [oil] = 0.2 and 0.5 wt % (respectively), suggesting that cellulose may have an influence on the miscibility of the IL and oil. Unfortunately, data for the solution with the highest cellulose concentration ([cellulose] = 2 wt %) with [oil] > 0.25 wt % was not reproducible due to the high viscosity of the solution and issues with filling the cuvette. This data has therefore been omitted from Figure 2. However, the absorbance at [oil] = 0.25 wt % was indeed the lowest for the 2 wt % cellulose solution, again indicating that the presence of cellulose may influence oil solubility in BmimAc.

## ^1^H NMR

### Pure BmimAc–Oil and BmimAc–Cellulose Solutions

**Figure 3:**
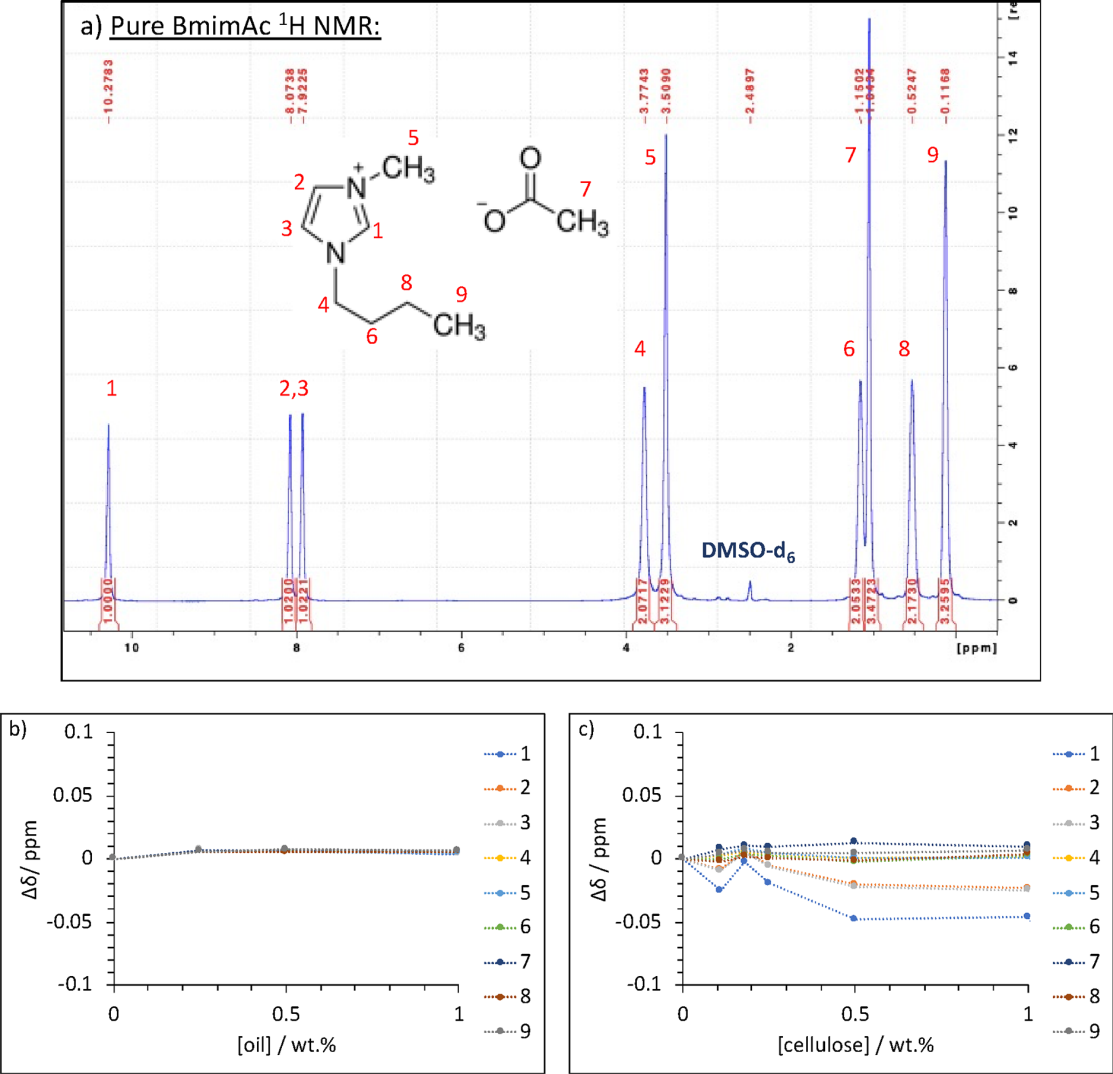
(a) High-field ^1^H NMR spectrum (400 MHz) of pure BmimAc (no oil), with peak assignments given in red for protons labeled 1–9. DMSO-*d*_6_ was used as a reference (δ = 2.5 ppm); (b) change in Δδ of protons 1–9 (BmimAc), as a function of oil concentration ([oil]); (c) change in Δδ of protons 1–9 (BmimAc), as a function of cellulose concentration ([cellulose]).

Mixtures of oil and BmimAc were prepared for high-field ^1^H NMR to investigate in more detail any interactions that might be occurring. From UV–vis analysis, we expect the maximum “solubility” of oil in BmimAc to be at [oil] ≃ 0.25 wt %, and therefore, we tested relatively low oil concentrations between 0 and 1 wt %, above which the oil appears to only temporarily be dispersing in BmimAc. Full assignment of the ^1^H NMR spectrum for MCT oil (Miglyol 812, made up of caprylic/capric triglycerides^64^) can be found in the Supporting Information (Figure S2). Since the amounts of oil added were very small, it was almost impossible to detect the corresponding peaks using NMR and therefore the chemical shift change (Δδ) of the BmimAc peaks was analyzed as a function of oil concentration, where δ of the pure IL peaks are used as a reference (for more details, see below Figure S2, Supporting Information). Figure 3a shows the spectrum of “oil-free” (pure) BmimAc with full peak assignments, corresponding to the different proton environments (H1–H9). Through analyzing Δδ as a function of the concentration of a component, one is able analyze the effect on specific interactions between the (Bmim)^+^ cation and/or the (OAc)^−^ anion, thus indirectly gaining information about component–IL interactions. This approach has been previously utilized for understanding cellulose dissolution in ILs,^10,13,68^ and here we apply the same principles to the addition of oil. It should be noted that an external reference DMSO-*d*_6_ was added to the NMR tube via a capillary (to ensure accuracy in determining minor peak shifts), since the presence of DMSO may also affect the BmimAc proton environments (see Materials and Methods for full details).

Figure 3b shows Δδ for BmimAc protons, as determined by ^1^H NMR, as a function of oil concentration (0–1 wt %). Only very minor changes were observed for BmimAc protons upon the addition of oil (Δδ < 0.01 ppm) and no further changes occurred as [oil] increased, suggesting that any interaction appears to be “saturated” above [oil] = 0.25 wt % (in agreement with UV–vis analysis). Interactions between BmimAc and oil are expected to be of hydrophobic nature and therefore would involve the aliphatic protons on the butyl chain of the cation (H9, H8, and H4) and the CH_3_ group on the acetate anion (H7). However, changes in chemical environments for all protons were very minor upon the introduction of oil and it appears that no strong interaction was present.

On the other hand, it is frequently reported that when cellulose dissolution occurs in imidazolium-based ILs, a significant change in the chemical environment is observed for H2, H3, and in particular, H1 (the most acidic proton), which forms a H-bond with the anion.^10,13,68^ H-bond interactions are generally much stronger than hydrophobic interactions and are estimated to be around 5 kcal/mol/pairing for the former (e.g., cellulose–cellulose) compared to 2 kcal/mol/residue for the latter.^26^Figure 3c shows Δδ for BmimAc protons as a function of cellulose concentration ([cellulose]), in comparison to oil. Once again, cellulose spectral bands were also not observed in the NMR spectra due to the relatively low concentrations studied, and therefore the small population of protons associated with the polymer,^10^ as well as the low mobility of cellulose molecules. However, a much greater Δδ is observed for the BmimAc peaks upon the addition of cellulose, which is widely understood to be a result of displacement of (Bmim)^+^ cations by cellulose hydroxyl groups, which form stronger H-bonds with (OAc)^−^ anions.^10^ Consequently, weakening of the cation–anion H-bond occurs as indicated by an increase in electron density around the aromatic protons (H1 in particular), leading to the upfield movement of δ (as indicated by a negative Δδ, Figure 3c). This has also been described as breakdown of IL clusters and ultimately ion pairs, which exist in the pure BmimAc solution but are disrupted when small amounts of cellulose are added (0.1–1 wt %).^69^ Unlike cellulose, oil appears to lack any significant interaction with the IL and there is negligible change to cation–anion H-bonding, suggesting that oil has a minimal effect on IL clusters. Therefore, it was speculated that at these concentrations, oil will have little or no effect on the ability of BmimAc to dissolve cellulose,^68^ unlike more polar solvents such as water.^4,40−43^

### Microscopic Properties of BmimAc–Oil–Cellulose Mixtures

**Figure 4:**
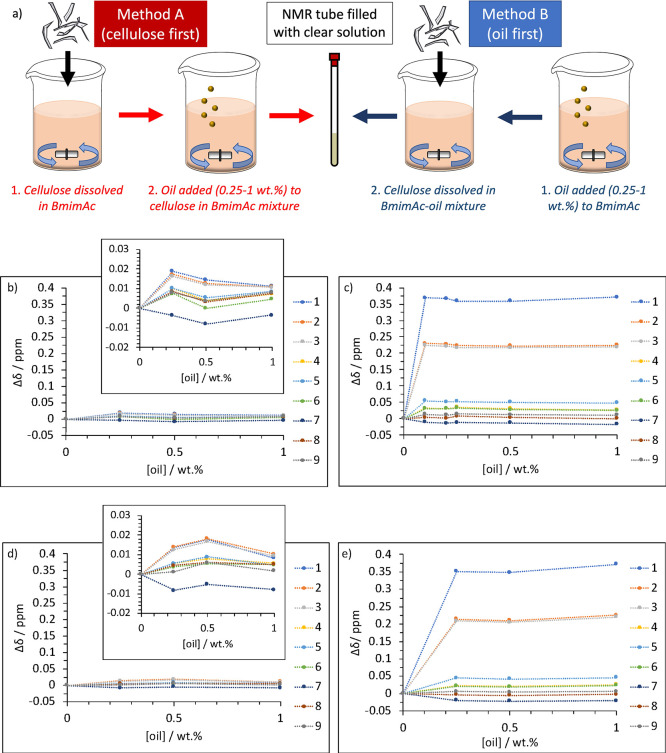
(a) Schematic showing methods for preparation of BmimAc–cellulose–oil mixtures, where oil was added either after (method A) or before (method B) complete cellulose dissolution; change in Δδ of protons 1–9 (BmimAc), as a function of oil concentration ([oil]), where (b) cellulose was dissolved before the addition of oil (method A) (inset shows a larger scale version); (c) cellulose was dissolved after the addition of oil (method B); (d,e) Δδ after 4 months of storage, for solutions prepared via methods A and B, respectively. In all cases, [cellulose] = 2 wt %.

In order to understand how the presence of oil may affect the cellulose-dissolving capacity of ILs, mixtures of all three components were prepared by two methods, either (A) by dissolving cellulose in BmimAc and subsequently adding oil or (B) by mixing the oil with BmimAc first and then adding cellulose (as shown by the schematic in Figure 4a). Figure 4b,c gives a comparison of Δδ for BmimAc protons in mixtures prepared by method A and method B, respectively. In both cases, oil was added after (method A) or before (method B) complete dissolution of 2 wt % cellulose (as indicated by the optically clear solutions). δ for each resonance in an “oil-free” 2 wt % cellulose–BmimAc solution was used as a starting reference value (for more details, see below Figure S2, Supporting Information), and thus Δδ represents the ppm change upon the addition of oil.^70^

**Figure 5:**
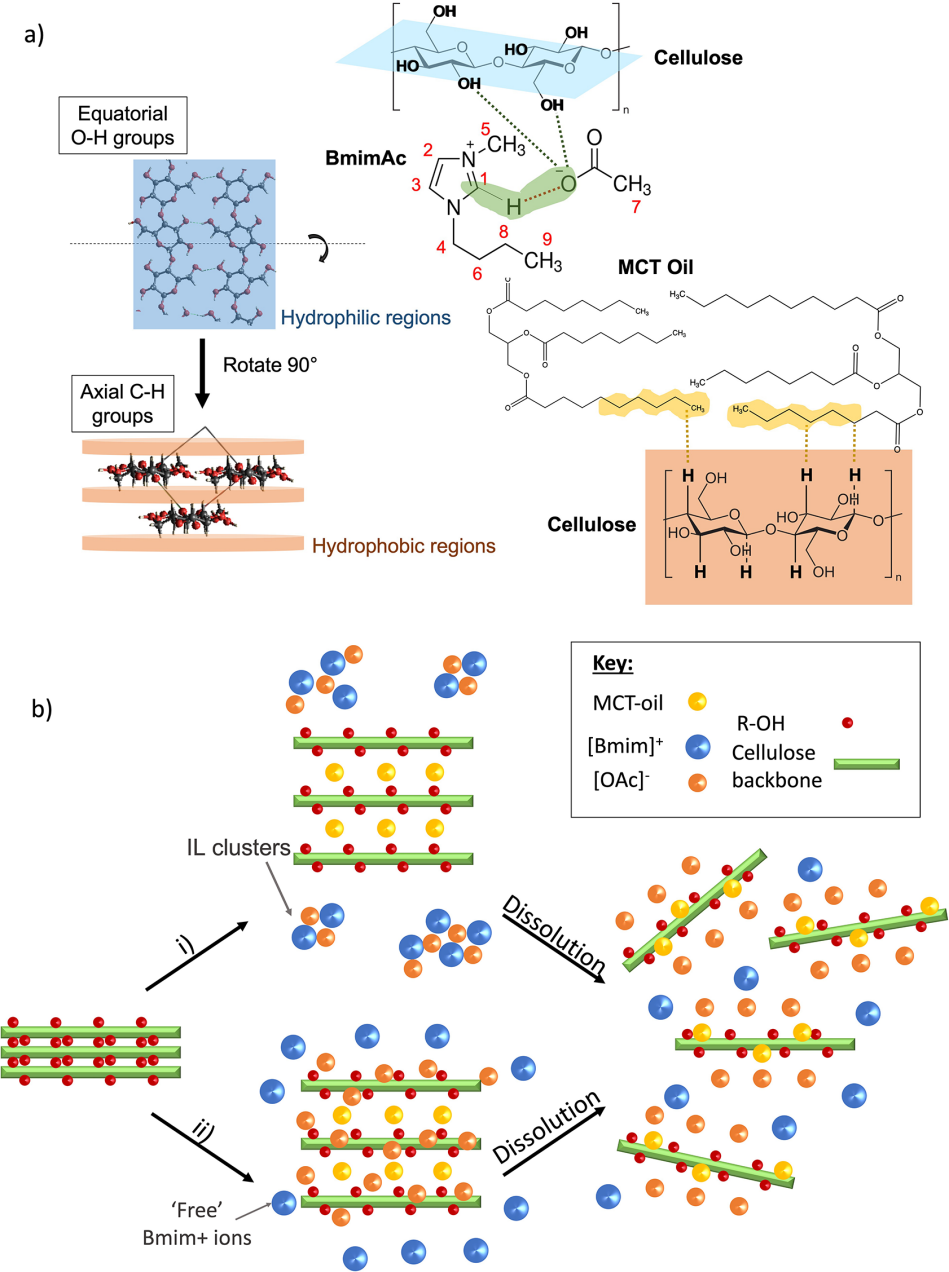
(a) Schematic showing hydrophobic/hydrophilic regions of cellulose (as described in ref (26)) and the suggested interactions; (b) schematic showing the two proposed possibilities for the dissolution mechanism of cellulose in BmimAc mixtures, with a cosolvent (oil).

From Figure 4b and particularly 4c, it is evident that the presence of cellulose affects Δδ for the BmimAc protons with the addition of oil. When oil was added after cellulose dissolution (method A, inset Figure 4b), the differences were relatively small but slightly more significant compared to BmimAc–oil mixtures in the absence of cellulose (Figure 3b), but when solutions were prepared by adding oil to BmimAc before cellulose dissolution (method B, Figure 4c), Δδ was at least 10 times higher for the acidic proton H1 (compared to method A). Resonances shifted downfield (increased in δ) with the addition of oil, which surprisingly is the opposite of what happens when cellulose is dissolved in BmimAc^69^ (where resonances shift upfield, Figure 3c). This downfield shift indicates a decrease in electron density around the aromatic protons and therefore strengthening of the cation–anion H-bond, since the aromatic protons (H1, H2, and H3) are much more affected by the addition of oil compared to the rest of the IL protons. Rather than oil interacting very weakly with the hydrophobic regions of the cation (as predicted in the absence of cellulose), we propose that it is now interacting preferentially with the hydrophobic plane of cellulose which weakens the cellulose–anion bond. The schematic in Figure 5a illustrates the amphiphilic character of cellulose and the potential IL–cellulose and oil–cellulose interactions present in cellulose–BmimAc–oil mixtures. The polarity of oil will also certainly have an effect on the strength of both hydrophilic and hydrophobic interactions and whether or not they even occur (as discussed previously); however, we were unable to verify this with lower polarity oils due their inability to solubilize in BmimAc. An oil with a very low polarity index may be “too hydrophobic” (non-polar) for any interaction with cellulose, in which case the cellulose–IL interaction would dominate.

The strengthening effect of the BmimAc H-bond was significantly greater when oil was added before cellulose (method B, Figure 4c) and we propose the following explanation: when cellulose is added via method B, the small amount of oil has already been solubilized as droplets in the IL and forms a kinetically stable system, due to the high viscosity of BmimAc. Cellulose is distributed throughout the solution and interacts with the oil droplets, orienting its hydrophobic planes toward the oil phase. This cellulose–oil interaction is favorable since cellulose has a greater hydrophobic surface area compared to Bmim^+^, which arises from the highly ordered axial orientation of C–H groups along cellulose chains and creates structural anisotropy^25^ (Figure 5a). Any weak cation–oil interactions are replaced with stronger cellulose–oil interactions, while the majority of the cellulose (dispersed in BmimAc) begins to dissolve. When oil is added after cellulose dissolution, the cellulose has already been molecularly dissolved, and therefore, the solution viscosity is much greater compared to pure BmimAc.^69^ Therefore, oil cannot be distributed as uniformly as in the absence of cellulose, and the volume of cellulose–oil interactions is reduced. A more thermodynamically stable state is preferred, where cellulose–IL interactions are maximized. As a result, the IL peaks are much less affected when oil is added to a solution of cellulose already in its molecularly dispersed state (Figure 4b), compared to a more ordered state (Figure 4c).

Notably, the δ values for the IL protons remarkably become comparable to δ values in the pure IL when oil is added to cellulose in BmimAc solution (method B, Figure 4c), suggesting that the chemical proton environments are similar to pure BmimAc (Figure S3). Despite the presence of the predicted cellulose–oil interaction, complete dissolution of cellulose still occurred in the oil–BmimAc solution when oil was added first (method B). This is most likely because such a small quantity of oil was added (<1 wt % compared to the IL), which has little or no effect on the dissolving capability of BmimAc and because the oil displays almost no interaction with the IL (as suggested by the above results). Furthermore, it has been reported that hydroxyl groups on cellulose interact more strongly with ILs compared to backbone protons (i.e., the hydrophobic planes)^71^ since the hydrophilic surface areas of cellulose are greater and in this case, the predicted oil–cellulose interaction does not involve hydroxyl groups and does not inhibit hydrophilic interactions between cellulose and the IL.

In order to understand whether solutions prepared via methods A and B are in an equilibrium state or that we were just observing a short time effect which results in differences, the NMR samples prepared via methods A and B were remeasured after storage for 1 month (Figure S4a,b) and 4 months (Figure 4d,e). Almost no change was observed for the solutions prepared via method A (Figure 4b,d), suggesting that no further oil–cellulose interactions developed within this time period and no cellulose precipitation occurred, the latter of which would cause the BmimAc H-bond to restrengthen. The oil, however, remained dispersed within the mixture and no separation occurred. We attribute this to the high viscosity of the cellulose–BmimAc medium and the low concentration of oil, resulting in solubilization of oil in the IL with long-term kinetic stability. While molecular cellulose does possess structural anisotropy and has been shown to arrange itself at the oil–IL interface over time,^72,73^ this does not appear to occur in solutions prepared via method A most likely due to the small volume of oil (and thus less opportunity for cellulose–oil interaction) and the high viscosity of the cellulose–IL solution. Therefore, the (Bmim)^+^ probably remained stacked within the cellulose planes and again, we suggest that the lack of significant cellulose–oil interaction is due to the molecular dispersion of cellulose in solution, which takes place before the oil is added (method A). Figure 4b,d also indicates that very little change was also observed microscopically for the solutions prepared via method B, when the oil was added before cellulose dissolution. Δδ was still more significant compared to method A after 4 months, and the downfield shift in the resonances for the aromatic protons H1, H2, and H3 remained the highest. Therefore, we conclude that the cellulose–oil interaction previously described was still present, and the system remained kinetically stable for this time period. (Bmim)^+^ cations and/or other cellulose molecules do not disrupt the existing cellulose–oil interactions, which is likely due to preferential exposure of the hydrophobic regions in cellulose to the oil, resulting in a significantly favorable interaction that aids long-term stability. In both cases, the solutions remained optically clear and therefore no cellulose precipitation was observed.

We propose that oil acts as a kind of cosolvent (as shown in the schematic, Figure 5b), and either “loosens” the cellulose structure (by initially “coating” the cellulose hydrophobic planes) before breakdown of IL clusters and penetration of IL molecules (Figure 5b, i) or helps to break up cation–anion pairs, freeing the anion and aiding dissolution (Figure 5b, ii). For the latter, a similar mechanism has been described for cellulose dissolution in DMSO–BmimAc mixtures where an increase in DMSO concentration led to a decrease in the viscosity, resulting in higher cellulose solubility.^74^ However, when we prepared solutions with lower (<0.25 wt %) and higher (>1 wt %) oil concentrations, no correlation was observed between Δδ and oil concentration (Figure S5a), suggesting that the role of oil is rather different from the role of DMSO (and other aprotic cosolvents). We hypothesize that oil penetrates between the hydrophobic cellulose planes, while the anion interacts with cellulose hydroxyl groups in the equatorial planes (represented by R–OH groups) through H-bonding, “freeing” the (Bmim)^+^ ions and followed by complete dissolution (Figure 5b, ii). Although the cellulose still appeared to be fully dissolved at higher oil concentrations (since no precipitation was observed), droplets of oil were visible under the microscope at [oil] = 2 wt % (Figure S5b), which probably led to the observed turbidity, and again suggests that there is little/no interaction between oil and BmimAc.

### Bulk Rheology of BmimAc–Oil–Cellulose Mixtures

**Figure 6:**
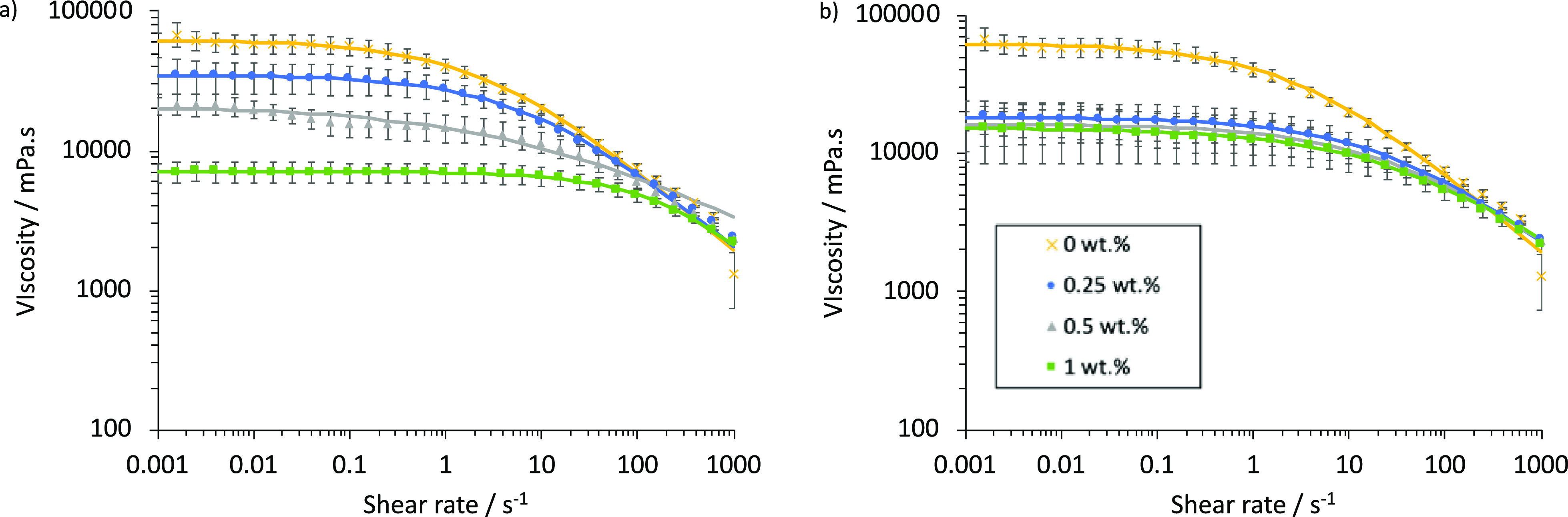
Flow curves at 25 °C for 2 wt % cellulose–BmimAc solutions with 0–1 wt % MCT oil added, where oil was added (a) after cellulose dissolution (method A) and (b) before cellulose dissolution (method B). [oil] = 0 wt % (yellow cross); 0.25 wt % (blue circle); 0.5 wt % (gray triangle); and 1 wt % (green square), legend shown on the bottom left of graph (b). Solid lines show fits to the cross-model equation (below Figure S6).

The bulk properties of BmimAc–oil–cellulose solutions were investigated in an attempt to gain further understanding of how the cosolvent (oil), BmimAc ions, and cellulose interact. Rheological analysis was chosen because the viscosity is very sensitive to changes in the degree of dissolution and the aggregation of the cellulose and therefore has the potential to reveal differences in solutions prepared via methods A and B as well as solutions with very minor differences in oil concentration. To the eye, the solutions appeared identical; however, as outlined in section “Microscopic Properties of BmimAc–Oil–Cellulose Mixtures,” microscopic differences were clearly observed when oil is added after or before cellulose to BmimAc. Figure 6a,b shows the flow curves for 2 wt % cellulose–BmimAc solutions with 0–1 wt % oil, prepared via methods A and B (respectively).

The viscosities of all solutions decreased upon the addition of oil, relative to the “oil-free” 2 wt % cellulose–BmimAc solution. In all cases, the mixtures showed shear-thinning behavior which is typical of cellulose–IL solutions.^75^ When oil was added after cellulose dissolution (method A, Figure 6a), the viscosity significantly decreased as the concentration of oil added increased and when [oil] = 1 wt %, the viscosity was 10 times lower compared to the “oil-free” solution. This is in agreement with the UV–vis and microscopy results and indicates that oil is simply dispersing in the IL mixture above [oil] = ca. 0.25 wt %. On the other hand, when oil was added before cellulose (Figure 6b), the viscosity initially decreased again by a larger magnitude but then showed a much less significant decrease upon higher oil addition. These observations again indicate that the order of oil addition has an effect on the interactions occurring in cellulose–BmimAc–oil mixtures and that oil may affect the mechanism of cellulose dissolution.

The zero-shear rate viscosities (η_0_) were obtained by fitting the viscosity curves to the cross model, given in the Supporting Information (below Figure S6), and relative viscosity (η_rel_ = η_0_/η_sol_, where η_sol_ is the zero-shear rate viscosity of pure BmimAc) was calculated. Figure S6 gives a plot of η_rel_ as a function of oil concentration, for the solutions prepared via methods A and B. A clear difference is observed between the two, despite the fact that the formulations of cellulose, oil, and BmimAc are identical and again it is clear that the order of addition has an effect on the interactions. For mixtures prepared via method A, η_rel_ decreased with increasing oil concentration, while for method B, η_rel_ was independent of [oil] (over this concentration range). This again indicates that when oil is added before cellulose, it disperses in BmimAc and then “sticks” to cellulose once it is introduced; thus, increasing the amount of oil hardly affects the relative viscosity. On the other hand, oil poorly disperses in the dissolved cellulose–BmimAc solutions (method A) and therefore viscosity decreases as a function of oil concentration.

### Mechanism of Cellulose Dissolution in BmimAc, in the Presence of Oil

It has been widely reported that dissolution of cellulose in BmimAc (in the absence of oil) is driven by H-bonding, with most mechanistic studies focusing on the hydrophilic interactions.^4,13,14^ While comparatively fewer reports pay attention to the hydrophobic interactions,^17,18^ it has been proposed that the cation interacts with hydrophobic regions on cellulose chains as a result of solvating the negatively charged anion–cellulose complex.^21,22^ Theoretical MD simulations have also indicated a cation–cellulose hydrophobic association, where the (Bmim)^+^ cation can stack between cellulose pyranose rings, and it was suggested that this compensates for the loss of interaction between the cellulose hydrophobic planes.^23,24^ Regardless of the type of interaction discussed and its importance, it is generally understood that hydrophilic and hydrophobic contacts between the IL and cellulose develop simultaneously rather than through a step-wise mechanism,^21^ and thus, ILs must breakdown both types of interaction in cellulose to achieve full dissolution.^25^ In contrast, studying the reverse process of dissolution (precipitation) has revealed that coagulation of cellulose from solution does in fact proceed via a step-wise mechanism. Isobe et al. used time-resolved synchrotron X-ray scattering to study precipitation of cellulose from aqueous alkali–urea^76^ and found that the initial process was driven by hydrophobic interactions. It was speculated that first, stacking of glucopyranoside rings occurred to form monomolecular sheets, which were subsequently lined up by H-bonding and formed cellulose crystallites. Several simulation studies have reported similar results, suggesting that H-bonds begin to form between molecular sheets after initial formation of the primary cellulose structure through van der Waals forces, driven by hydrophobic association.^77−79^

**Figure 7:**
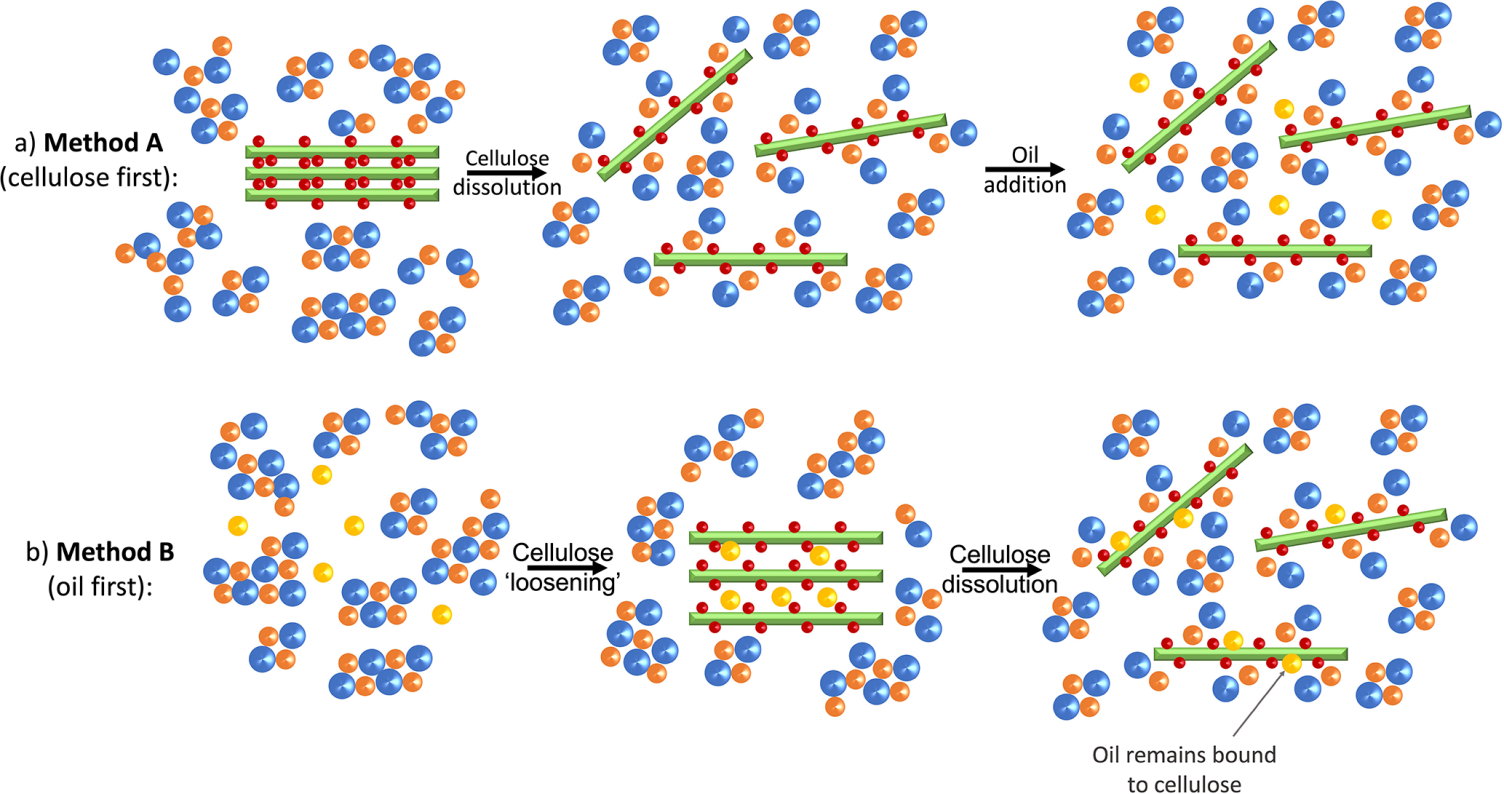
Schematic showing cellulose dissolution in the presence of oil: (a) when cellulose is added first and dissolution occurs, cosolvent (oil) is added after (method A); (b) when cosolvent (oil) is added to BmimAc first, followed by cellulose (method B), resulting in an oil–cellulose interaction. Each component is represented in the same way as Figure 5b.

Considering the mechanisms for both dissolution (including in the presence of cosolvents, Figure 5b) and coagulation, we propose two possible roles for oil during dissolution of cellulose in BmimAc depending on the order of its addition (method A or B, illustrated in Figure 7a,b, respectively). If cellulose is dissolved first (method A), disruption of its crystalline structure occurs with the development of both hydrophilic and hydrophobic associations between BmimAc and cellulose. Although hydrophobic interactions between the cation and the hydrophobic regions of cellulose are expected to be weak,^17,18,21,23^ when oil is subsequently introduced it can barely displace the cation. Furthermore, cellulose is already molecularly dispersed and no longer possesses a long-range order, resulting in shorter chain lengths^72^ and a smaller axial hydrophobic surface area available for oil interaction (Figure 7a). However, when the cosolvent (oil) is introduced before cellulose (method B), it disperses in BmimAc with little or no interaction and does not make the cations or anions any less available for cellulose. Upon introduction of cellulose, the oil may “coat” its intact axial hydrophobic planes and penetrate between the glucopyranose rings by a stacking interaction. Since the amount of oil is small relative to the amount of IL, the (OAc)^−^ anions can still sufficiently disrupt inter/intramolecular cellulose–cellulose interactions and form H-bonds with the equatorial hydroxyl groups, resulting in complete dissolution (Figure 7b). The apparent “re-strengthening” of the cation–anion H-bond observed (Figure 4c) is due to the higher volume of “cellulose-free” (Bmim)^+^ ions, liberated by the presence of cosolvent (oil) molecules between cellulose chains which “compete” with the (Bmim)^+^ cations for hydrophobic association to cellulose.

As well as considering the state of the cellulose at the time of oil addition and its effect on the oil–cellulose interaction, the number of components present at one time in the solution may also be a key factor. Kuzmina et al. reported that when formic acid (FA) was introduced as a cosolvent before cellulose dissolution, a “competition effect” was observed where FA and BmimAc competed for interaction with the cellulose.^53^ Although the FA caused an upfield shift of the (Bmim)^+^ protons (unlike the downfield shift observed in this case with oil), the same principle can be applied. For method B (Figure 7b), cellulose is introduced to oil and BmimAc at the same time, while for method A (Figure 7a), cellulose–IL interactions have already developed before oil is introduced. Therefore, we propose that oil must replace the (Bmim)^+^ ions in the glucopyranose stacks^24^ for any cellulose–oil interaction to occur in method A, as opposed to oil and (Bmim)^+^ “competing” for cellulose interaction during dissolution (method B), and consequently the cellulose–oil interaction and the observed Δδ are greater in the latter case.

Compared to other cosolvents commonly employed in cellulose dissolution in ILs (e.g., DMSO), MCT oil is a relatively “polar” oil with three carbonyl groups (making it a weak HBD). Therefore, the oil displays a lower miscibility with BmimAc and can only be added in very small quantities since above ca. 1 wt %, oil is only temporarily dispersing in the IL. Microscopic studies of dissolution in BmimAc with various cosolvents showed that when cellulose was preswollen in 5 wt % DMSO, Δδ was effectively 0, indicating that the cosolvent had little effect on cation–anion bonding.^53^ Despite this, DMSO influenced cellulose dissolution and the authors attributed this to an initial “loosening” of the cellulose structure by DMSO (similar to what we described in Figure 7b), which was then replaced by the main IL solvent most probably entirely, judging by the negligible change in δ of BmimAc. In our case, we suggest that the oil remains associated with cellulose in the presence of BmimAc, since neither the cation nor the anion cannot “outcompete” the oil for hydrophobic association with cellulose. This explains the positive Δδ (downfield shift) and the strengthening of the cation–anion H-bond that we described, which is a result of oil first loosening the cellulose structure and then the remaining stacked within the hydrophobic planes, while a smaller volume of (Bmim)^+^ ions are locked within the cellulose structure and thus are free for cation–anion H-bonding.

It should also be mentioned that the perceived “re-strengthening” of the cation–anion H-bond could be explained by partial disruption of weakly bound IL clusters by the oil, which would increase the number of cation–anion ion pairs and thus increase the strength of the H-bond interaction.^80^ However, we rule out this explanation first because the concentration of oil added is too low to have a significant effect and second because one would expect the same Δδ regardless of the order of oil addition, while Figure 4b,c clearly displays a significant difference between methods A and B. In addition, we expect any oil–IL interactions to be much weaker than H-bond interactions in IL molecule clusters.

### BmimAc–Oil–Cellobiose Mixtures

**Figure 8:**
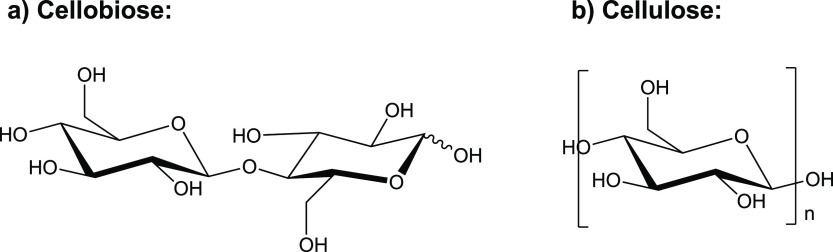
Chemical structures of (a) cellobiose and (b) repeat unit of cellulose.

To further investigate the importance of the cellulose state for the development of cellulose–oil interactions, experiments were carried out using an alternative carbohydrate cellobiose as a cellulose model.^13^ Cellobiose, like cellulose, has the same β-1,4-glycosidic linkage between two glucopyranose units (Figure 8a) but does not have the same repeating (polymeric) structure (Figure 8b), and therefore, the viscosity of cellobiose–BmimAc solutions can be between one and three times lower compared to cellulose–BmimAc solutions.^81^ This is advantageous because larger concentrations of cellobiose can be dissolved and analyzed, which in our case may enhance the cellobiose–oil interactions (and Δδ). It is also commonly used as a cellulose model for simulation studies, due to the limit of computational power.^82^

Cellobiose–BmimAc solutions were prepared with 0–1 wt % MCT oil via method B (oil added before), either with 2 wt % cellobiose for comparison to the cellulose–BmimAc solutions or with a higher concentration of 15 wt % cellobiose, to maximize the possibility of detecting any oil–cellobiose interaction. In both cases, δ for each resonance in an “oil-free” cellobiose–BmimAc (either 2 or 15 wt %) solution was used as a starting reference value (for more details, see below Figure S2 Supporting Information). Table 1 gives a comparison between Δδ for the BmimAc protons as a function of [oil] for 2 wt % cellulose/cellobiose–BmimAc solutions.

**Table 1:** Comparison of the Changes to Chemical Shift (Δδ) for H1 of BmimAc in 2 wt % Cellulose/Cellobiose–BmimAc–Oil Solutions, Prepared by Adding Oil before Cellulose/Cellobiose (Method B)^a^

concentration of oil/wt %	Δδ 2 wt %cellulose/ppm	Δδ 2 wt %cellobiose/ppm
0	–0.4142	–0.0364
0.25	0.3594 (ca. ∼12×)^b^	–0.0305
0.5	0.3596 (ca. ∼13×)^b^	–0.0277
1	0.3709 (ca. ∼47×)^b^	–0.0079

a For 0 wt % oil solutions, Δδ is calculated with pure BmimAc as a reference, and, for 0.25–1 wt % oil solutions, Δδ is calculated with the corresponding 2 wt % cellulose/cellobiose–BmimAc solution as a reference.

b Relative to the reference value.

Almost no effect was observed for any of the BmimAc protons in cellobiose–BmimAc solutions with the addition of oil (Δδ < 0.01), at both the higher (15 wt %, Figure S7a) and lower (2 wt %, Figure S7b) concentrations of cellobiose analyzed. This suggests that oil does not play a role in cellobiose dissolution whatsoever, unlike for cellulose, and this difference is clearly displayed in Table 1, where Δδ for the most acidic proton (H1) is compared. We argue that this highlights the importance of the long-range order in the cellulose structure and the presence of the hydrophobic planes,^83^ which are necessary for a significant cellulose–oil interaction and are not present in cellobiose. These results also confirm that the more significant Δδ observed for cellulose–BmimAc–oil solutions prepared by method B (Figure 4c) must be due to a hydrophobic interaction rather than hydrophilic, since cellobiose is still capable of forming H-bonds with components in solution. In fact, cellobiose is expected to have a greater capacity for H-bonding compared to cellulose due to the greater number of hydroxyl groups per glucose unit (*N*). A previous study comparing cellulose, cellobiose, and glucose (*N* = 3, 4, and 5, respectively) in ILs has shown that the associated fraction (α) is an important parameter to consider when comparing carbohydrates,^81^ where α is defined as follows
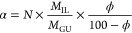
1where *N* = number of OH groups “per glucose unit” (4 for cellobiose and 3 for cellulose); *M*_IL_ = mass of the IL; *M*_GU_ = mass of “glucose units” (171 and 162 g mol^–1^ for cellobiose and cellulose, respectively); and ϕ = wt % of the carbohydrate. Therefore, α gives representative value for the fraction of IL molecules involved in dissolving “units of glucose,” and thus allows comparison between the carbohydrates. For 2 wt % cellulose and cellobiose solutions, α = 0.075 and 0.095, respectively, while for 15 wt % cellobiose solutions, α = 0.818, suggesting potential for a larger volume of hydrophilic cellobiose–IL associations compared to cellulose–IL. This was particularly evident for the 15 wt % cellobiose–IL solutions, where broadening of the peaks was observed in ^1^H NMR spectra (Figure S8), most probably due to exchange of the cellobiose hydroxy protons with the acidic protons in the (Bmim)^+^,^13^ while Δδ values displayed in Figure S7a provide no evidence for a hydrophobic cellobiose–oil interaction. This strongly suggests that the mechanism of cellobiose dissolution in BmimAc involves hydrophilic interactions alone rather than both hydrophilic and hydrophobic as in the case of cellulose, and that the highly orientated repeat structure of cellulose is responsible for its hydrophobicity. Again, it appears that the cellulose structure must be intact for significant oil–cellulose interaction to occur (method B, see section “Mechanism of Cellulose Dissolution in BmimAc, in the Presence of Oil”).

## Conclusions

In this work, we highlight the importance of hydrophobic interactions in cellulose dissolution in ILs, which evidently must be disrupted between cellulose molecules in order to achieve complete dissolution. Full dissolution of cellulose was achieved by BmimAc in the presence of MCT oil, when it was added to the IL both before and after the cellulose. However, we report that the order of oil addition has an effect on the interactions in the solutions and we observed a significant increase in Δδ (downfield shift) for BmimAc–cellulose peaks when MCT oil was added before cellulose. A more significant decrease in viscosity was also observed with increasing oil concentration when oil was added after cellulose, as opposed to the former case. We rationalize these differences by considering the solution state of cellulose: when the oil is introduced first, a hydrophobic interaction develops between the intact hydrophobic plane of cellulose and oil. However, when cellulose is introduced first, it is molecularly dispersed when oil is added and there is no significant interaction. The same effect was not observed for cellobiose (in BmimAc–oil solutions), which is commonly used as a model for cellulose dissolution studies, suggesting that the structural anisotropy of cellulose is important. We also highlight that this indicates differences between the mechanisms of cellulose and cellobiose dissolution in ILs.

Furthermore, the cellulose–oil interaction that we described is similar to a “pre-swelling” stage described for other cosolvents in ILs (for example DMSO), and we propose that the oil can act as a type of cosolvent, penetrating between the cellulose glucopyranose rings and interacting with the axial hydrophobic planes of cellulose. Over time, the cellulose–oil interaction remains and is not “outcompeted” by BmimAc–cellulose or cellulose–cellulose interactions, resulting in a stable system and thus a potential route for trapping oil within the cellulose structure (upon coagulation). This work provides further insights into the mechanism of cellulose dissolution in ILs, the importance of hydrophobic interactions, and the effect of non-polar cosolvents, which is important in the design of novel ILs for efficient cellulose dissolution.

## Supporting Information Available

Details of Vitacel cellulose properties; UV–vis spectrum of oil–BmimAc mixtures; NMR spectrum of MCT oil; details on calculating Δδ for NMR spectra; NMR spectra for cellulose–oil–BmimAc and pure BmimAc; Δδ for mixtures after 1 month of storage; Δδ and optical micrograph of mixtures with 2 wt % oil; η_rel_ as a function of oil concentration; Δδ for cellobiose–oil–BmimAc mixtures; and NMR spectrum of cellobiose–BmimAc (PDF)

## Abbreviations

BmimAc: 1-butyl-3-methyl imidazolium acetate
DMF: dimethyl formamide
DMSO: dimethyl sulfoxide
FA: formic acid
H-bonding: hydrogen bonding
HBD: hydrogen bond donor
IL: ionic liquid
MCT: medium-chain triglyceride
MD: molecular dynamics
MGU: mass of glucose unit
MIL: mass of the ionic liquid
NMR: nuclear magnetic resonance spectroscopy
ppm: parts per million
UV–vis: ultraviolet–visible
W/O: water-in-oil
