# Supplementary material for: Effect of Oil on Cellulose Dissolution in the Ionic Liquid 1-Butyl-3-methyl Imidazolium Acetate
Source: ACS Omega. 2022 Oct 12;7(42):37532–45. doi: 10.1021/acsomega.2c04311 (PMC9608373; doi:10.1021/acsomega.2c04311)
Supplement: Supplementary file 1 — ao2c04311_si_001.pdf [file ao2c04311_si_001.pdf]

Supporting Information

The effect of oil on cellulose dissolution in the ionic liquid 1-butyl-3-methyl imidazolium acetate

*Katherine S. Lefroy[1], Brent S. Murray[1], and Michael E. Ries[2]**

1 School of Food Science and Nutrition, University of Leeds, Leeds, LS2 9JT, UK

2 School of Physics and Astronomy, University of Leeds, Leeds, LS2 9JT, UK

Keywords: Ionic Liquid, Cellulose, Rheology, NMR, Oil

fskl@leeds.ac.uk, B.S.Murray@leeds.ac.uk, *M.E.Ries@leeds.ac.uk

Table S1. Vitacel Powdered Cellulose L 00 (V-cell): information provided by the manufacturer (J. Rettenmaier & Söhne GmbH & Co. KG)

	**Vitacel Powdered Cellulose L 00**
Colour	White
Structure	Fibres
Dietary Fiber Content	~98%
Bulk Density	~165 g/L
Fiber length	120 μm
Water binding capacity	525 %
Oil absorption	410 %
Degree of polymerization (DP)	730-830[1]

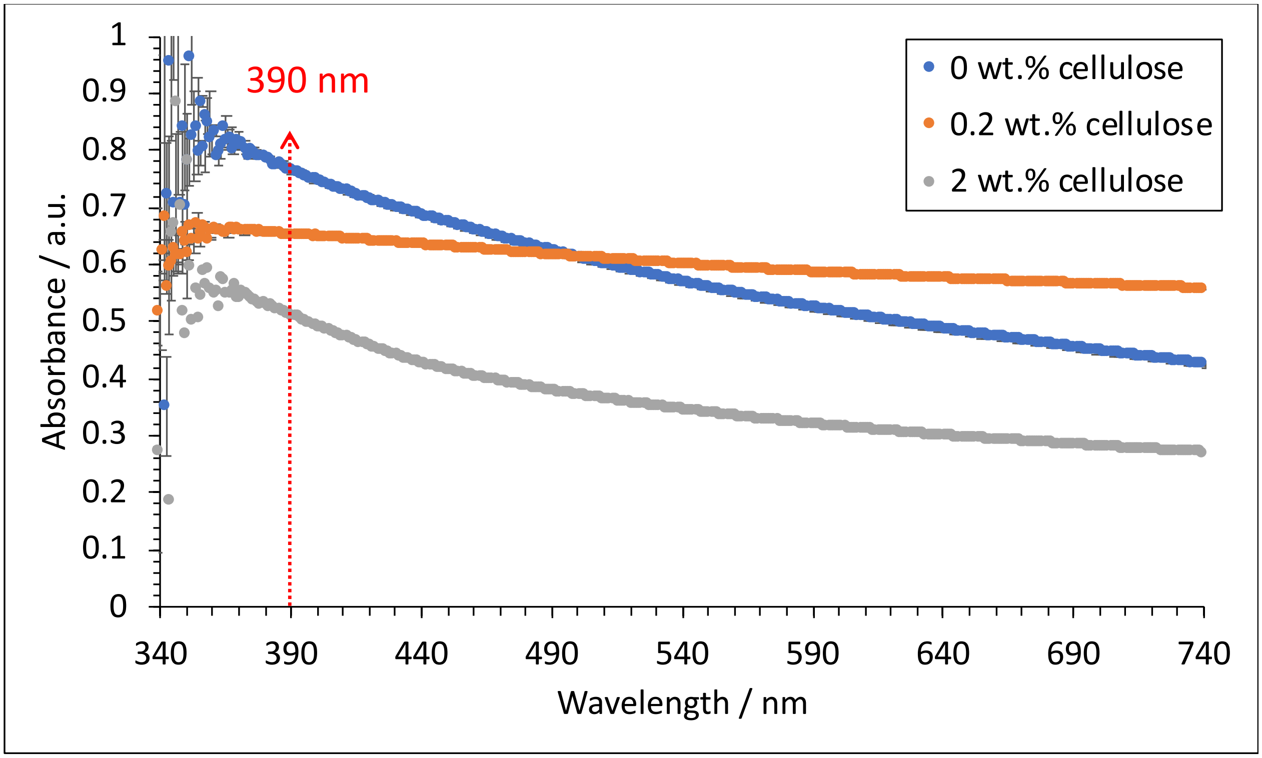

Figure S1. UV-vis absorbance spectrum (measured from λ = 180-800 nm) of an oil-BmimAc solution (blue), 0.2 wt.% cellulose-BmimAc-oil solution (orange) and 2 wt.% cellulose-BmimAc-oil solution (grey), where in each case, [oil] = 0.25 wt.%. Reference blanks of pure BmimAc, 0.2 wt.% cellulose-BmimAc and 2 wt.% cellulose-BmimAc solutions were used for each respective cellulose-BmimAc-oil solution. Error bars are shown but are covered by the symbols above λ ≃360 nm, absorbance data below λ = 340 nm is omitted due to the high level of noise

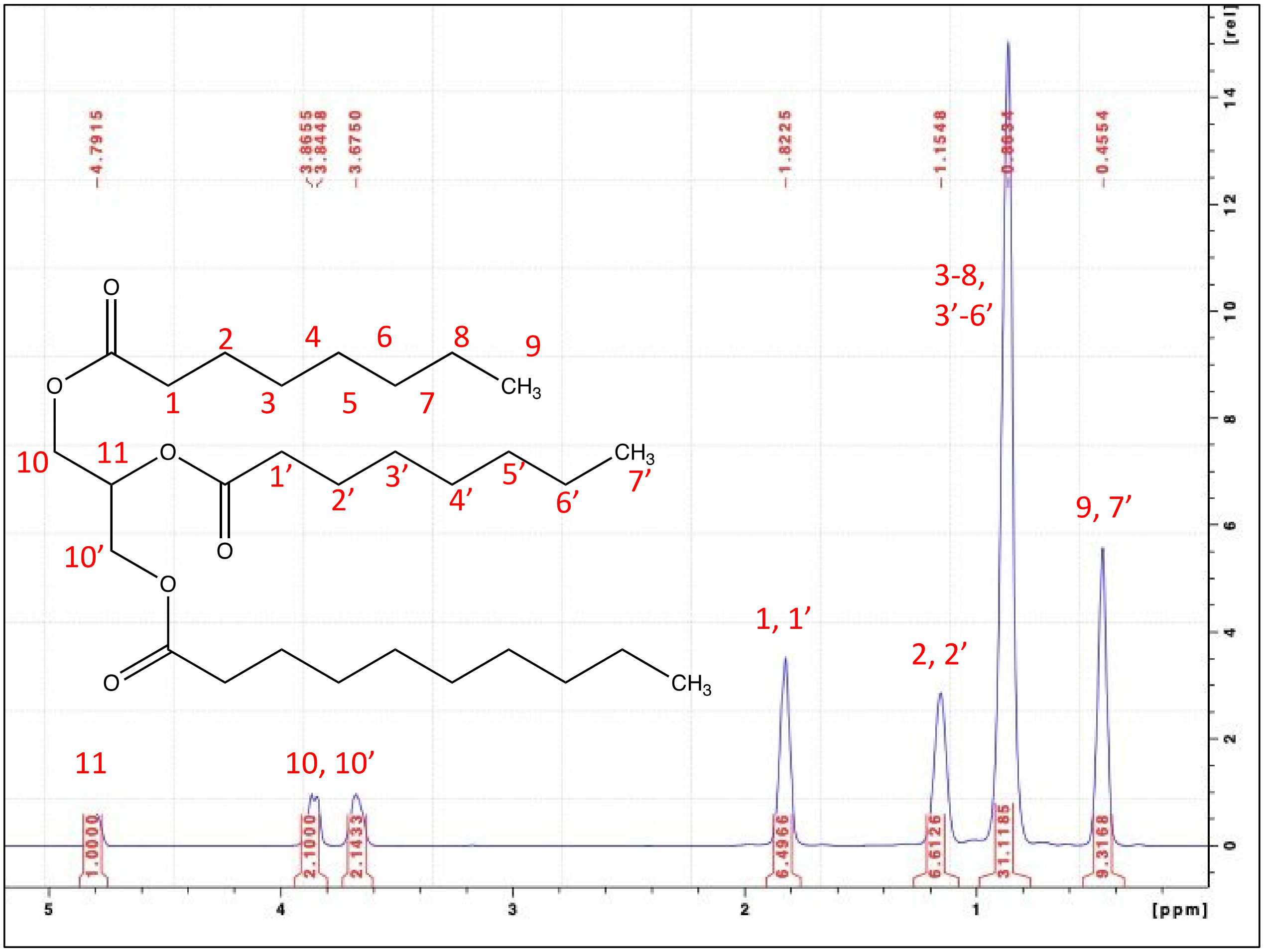

Figure S2. ^1H NMR (400 MHz) spectrum of MCT-oil (Miglyol 812), with peak assignments to capric/caprylic acid shown in red in agreement with[2]

Details on calculating Δδ for cellulose-BmimAc, cellulose-BmimAc-oil and cellobiose-BmimAc-oil solutions:

For pure BmimAc-oil solutions, the δ values of the peaks for the pure BmimAc were used as a reference. Therefore, the change in δ (Δδ) with [oil] is given as follows:

$$\Delta\delta = \delta_{[oil]} - \delta_{[BmimAc]}$$

Where $\delta_{[oil]}$ represents δ of peaks in the BmimAc-oil samples and $\delta_{[BmimAc]}$ represents δ of the corresponding reference peak, in the pure IL.

For cellulose-BmimAc-oil solutions where [cellulose] = 2 wt.%, the δ values of the peaks for the 'oil-free' 2 wt.% cellulose-BmimAc solution were used as a reference. Therefore, the change in δ (Δδ) with [oil] is given as follows:

$$\Delta\delta = \delta_{[oil]} - \delta_{[C_{BmimAc} = 2\ wt.\%]}$$

Where $\delta_{[oil]}$ represents δ of peaks in the BmimAc-oil samples and $\delta_{[BmimAc]}$ represents δ of the corresponding reference peak, in the 'oil-free' 2 wt.% cellulose-BmimAc solution.

For cellobiose-BmimAc-oil solutions where [cellobiose] = 15 wt.%, the δ values of the peaks for the 'oil-free' 15 wt.% cellobiose-BmimAc solution were used as a reference. Therefore, the change in δ (Δδ) with [oil] is given as follows:

$$\Delta\delta = \delta_{[oil]} - \delta_{[C_{BmimAc} = 15\ wt.\%]}$$

Where $\delta_{[oil]}$ represents δ of peaks in the BmimAc-oil samples and $\delta_{[BmimAc]}$ represents δ of the corresponding reference peak, in the 'oil-free' 15 wt.% cellobiose-BmimAc solution.

For cellobiose-BmimAc-oil solutions where [cellobiose] = 2 wt.%, the δ values of the peaks for the 'oil-free' 2 wt.% cellobiose-BmimAc solution were used as a reference. Therefore, the change in δ (Δδ) with [oil] is given as follows:

$$\Delta\delta = \delta_{[oil]} - \delta_{[C_{BmimAc} = 2\ wt.\%]}$$

Where $\delta_{[oil]}$ represents δ of peaks in the BmimAc-oil samples and $\delta_{[BmimAc]}$ represents δ of the corresponding reference peak, in the 'oil-free' 2 wt.% cellobiose-BmimAc solution.

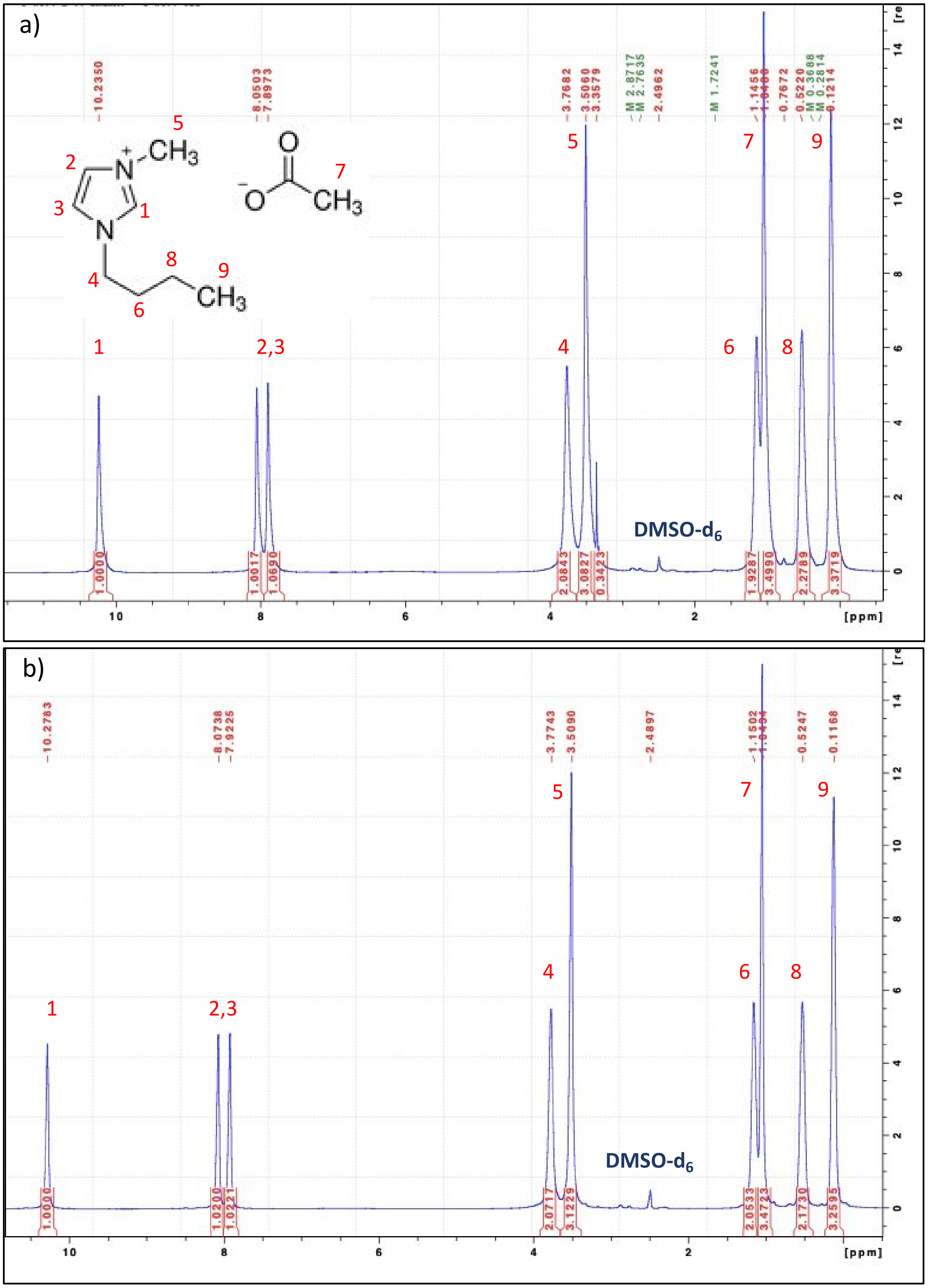
a)
DMSO-d6
10.2350
8.0503
7.8973
3.7682
3.5060
3.3579
2.4962
1.1456
0.7672
0.5220
0.1214
1.0000
1.0017
1.0690
2.0843
3.0827
0.3423
1.9287
3.4990
2.2789
3.3719
[ppm]
b)
DMSO-d6
10.2763
8.0738
7.9225
3.7743
3.5090
2.4897
1.1502
0.5247
0.1168
1.0000
1.0200
1.0221
2.0717
3.1229
2.0533
3.4723
2.1730
3.2595
[ppm]

Table S1. Chemical shift values (δ) for cellulose-BmimAc-oil and pure BmimAc solutions, corresponding to figures S4a and S4b respectively

Proton number	δ for S3a, ([cellulose] = 2 wt.%; [oil] = 1 wt.%) / ppm	δ for S3b, (pure BmimAc) / ppm
1	10.235	10.2783
2	8.0503	8.0738
3	7.8973	7.9225
4	3.7682	3.7743
5	3.5060	3.5090
6	1.1456	1.1502
7	1.0488	1.0434
8	0.5220	0.5247
9	0.1214	0.1168

Figure S3. ^{1}H NMR spectrum for a) cellulose-BmimAc-oil solution prepared via method B, ([cellulose] = 2 wt.%; [oil] = 1 wt.%) and b) pure BmimAc, highlighting the similarity between δ values (Δδ ≃ 0), suggesting that the addition of the oil 're-strengthens' the cation-anion H-bond and the proton resonances return to those of the pure BmimAc solution. The peak at Δδ ≃ 2.5 ppm corresponds to the external reference, DMSO

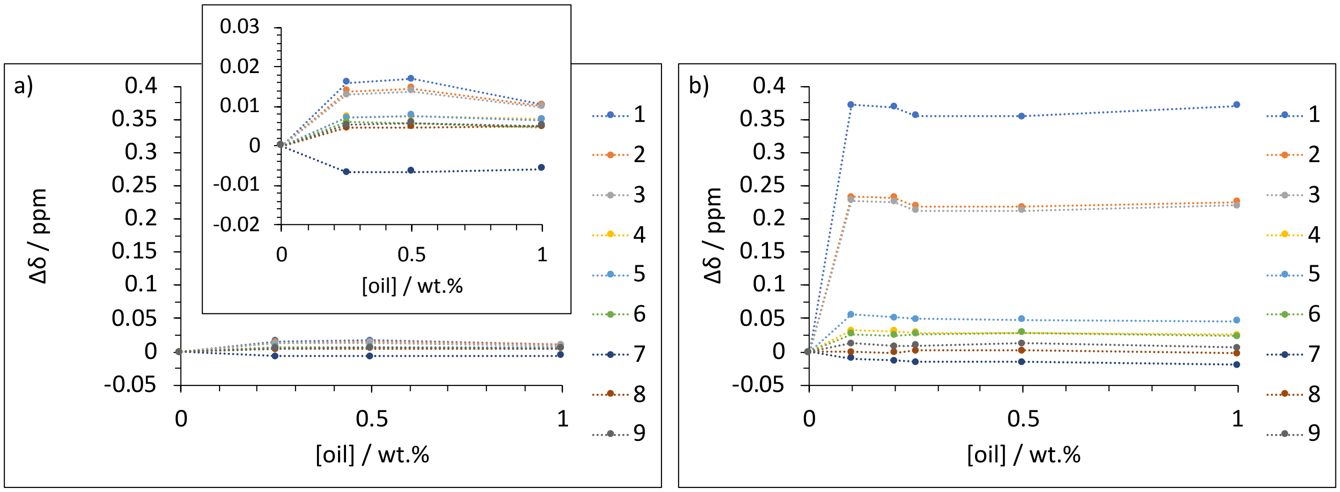

Figure S4. Change in chemical shift values (Δδ) of protons 1-9 (BmimAc) after 1 month storage, as a function of oil concentration ([oil]), where a) cellulose was dissolved before the addition of

oil (method A) and b) cellulose was dissolved after the addition of oil (method B). In both cases, [cellulose] = 2 wt.%

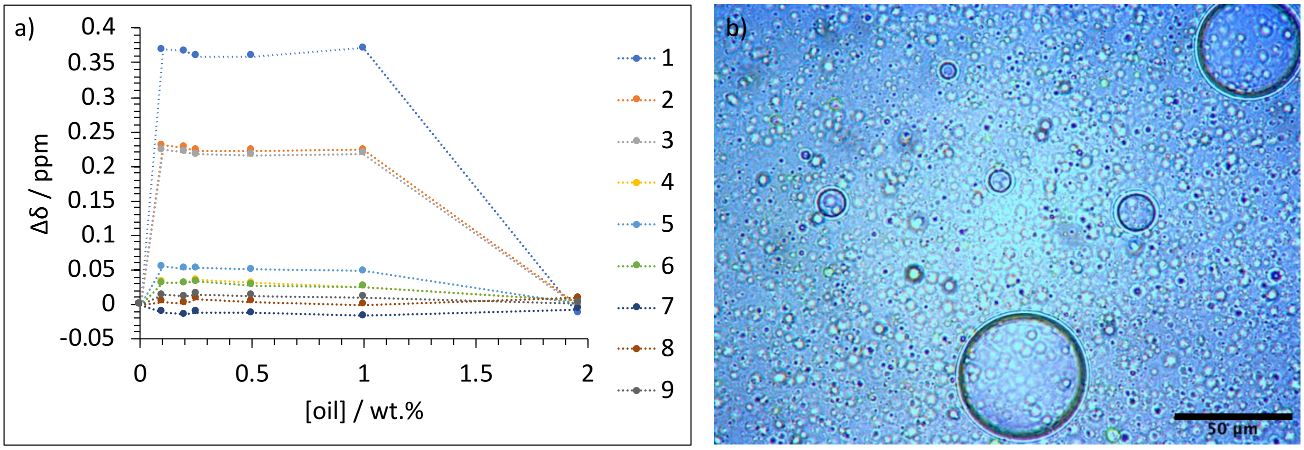

Figure S5. a) Change in chemical shift values (Δδ) of protons 1-9 (BmimAc), as a function of oil concentration ([oil] = 0-2 wt.%) for [cellulose] = 2 wt.%, where cellulose was dissolved after the addition of oil (method B); b) optical micrograph of cellulose-BmimAc-oil solution, where [cellulose] = 2 wt.% and [oil] = 2 wt.%

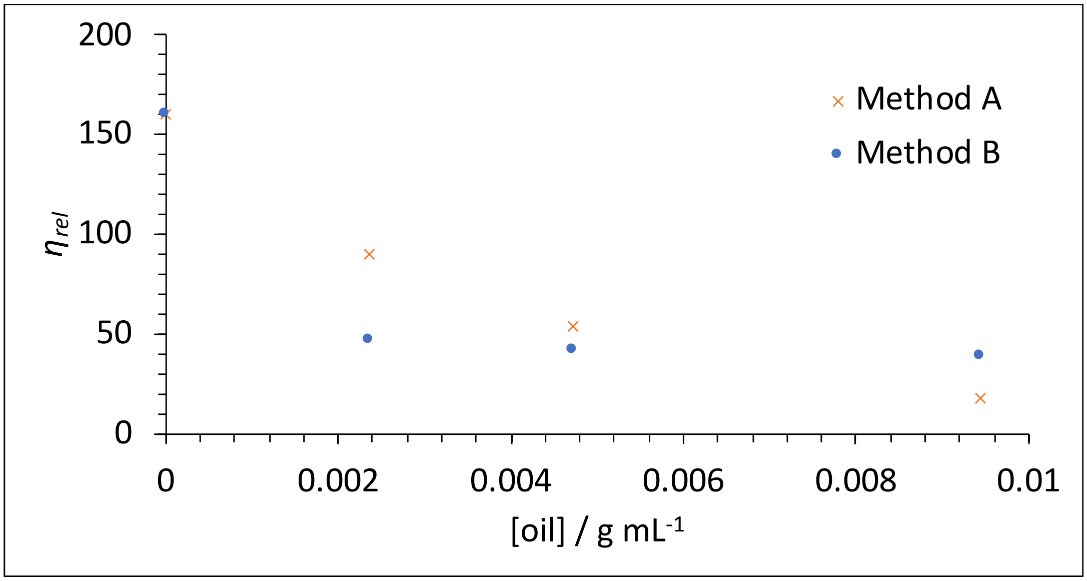

Figure S6. Relative viscosity (η_{rel}) as a function of [oil], where the zero-shear rate viscosities (η_0) were determined by fitted the flow curves given in figure 6 to the Cross-model equation:

$$\eta_{\dot{\gamma}} = \eta_{\infty} + \frac{\eta_0 - \eta_{\infty}}{1 + (C\dot{\gamma})^m}$$ **Equation S1**

Where η_0 = zero-shear rate viscosity; η_∞ = infinite-shear rate viscosity; C = Cross time constant and m = (Cross) rate constant

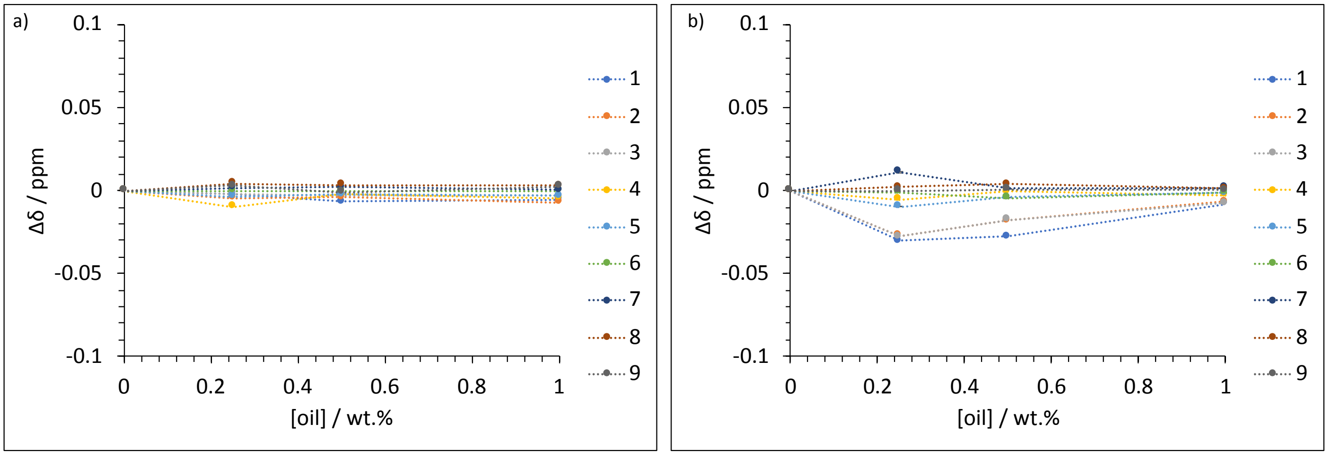

Figure S7. Change in chemical shift values (Δδ) of protons 1-9 (BmimAc), as a function of oil concentration ([oil] = 0-1 wt.%) for a) [cellobiose] = 15 wt.%, and b) [cellobiose] = 2 wt.%, where cellobiose was dissolved after the addition of oil (method B)

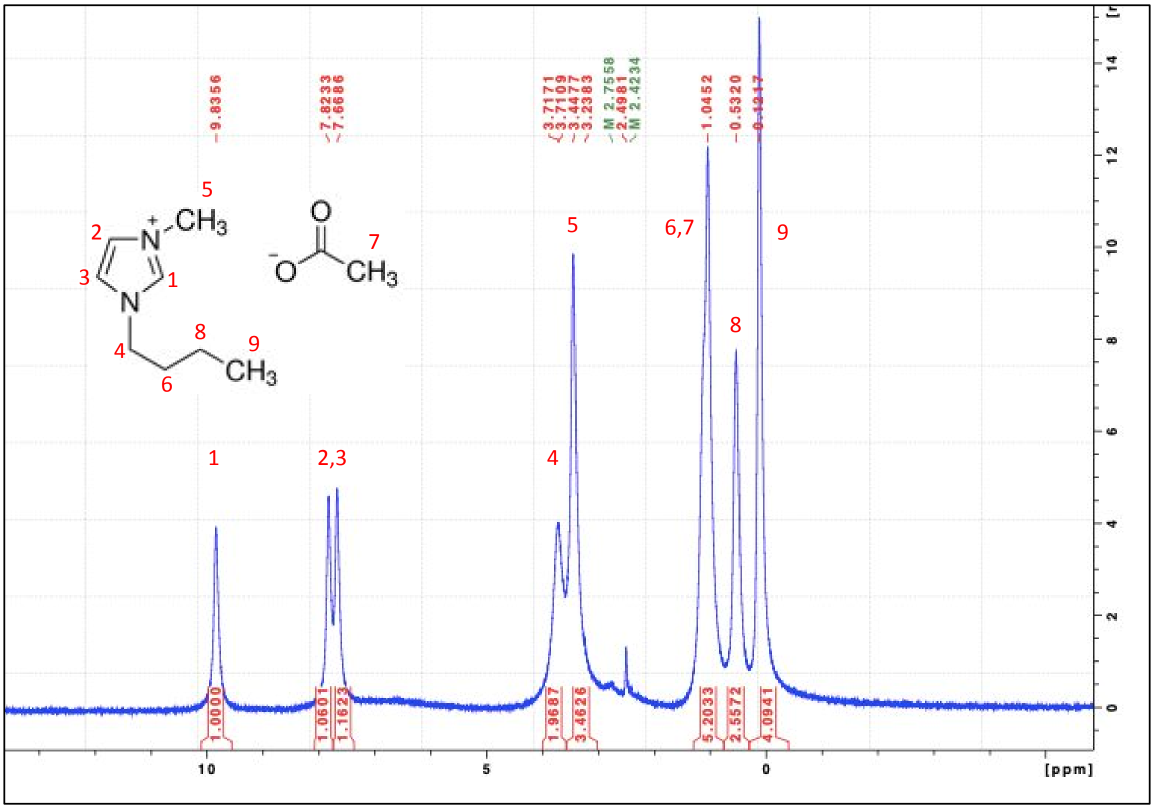

Figure S8. ^{1}H NMR spectrum (400 MHz) for 15 wt.% cellobiose dissolved in BmimAc, with peak assignments shown in red

REFERENCES

(1) Lefroy, K. S.; Murray, B. S.; Ries, M. E. Rheological and NMR Studies of Cellulose Dissolution in the Ionic Liquid BmimAc. *J. Phys. Chem. B* **2021**, *125*, 8205–8218.

(2) Yan, X.; Alcouffe, P.; Sudre, G.; David, L.; Bernard, J.; Ganachaud, F. Modular Construction of Single-Component Polymer Nanocapsules through a One-Step Surfactant-Free Microemulsion Templated Synthesis. *Chem. Commun.* **2017**, *53*, 1401–1404.
